# Clinical performance of the pseudo-non diffracting beam Toric EDOF intraocular lens: visual function, rotational stability, and quality of life

**DOI:** 10.3389/fmed.2025.1666607

**Published:** 2025-10-16

**Authors:** Erika Bonacci, Marco Anastasi, Camilla Pagnacco, Francesca Barzaghi, Arianna Serraiotto, Francesca Pilati, Adriano Fasolo, Emilio Pedrotti

**Affiliations:** ^1^Ophthalmology Clinic, Department of Engineering for Innovation Medicine, University of Verona, Verona, Italy; ^2^Ophthalmology Clinic, Department of Surgery, Dentistry, Maternity and Infant, University of Verona, Verona, Italy

**Keywords:** Toric EDOF IOL, patient satisfaction, quality of life, contrast sensitivity, cataract extraction, intraocular lenses

## Abstract

**Purpose:**

To evaluate visual acuity, refractive outcomes, rotational stability, and patient-reported satisfaction 3 months after bilateral implantation of the Lucidis Toric extended-depth-of-focus (EDOF) intraocular lens in cataract patients with 1.00–3.00 D of regular corneal astigmatism.

**Methods:**

Prospective, single-arm study of 25 patients (50 eyes) undergoing phacoemulsification with bilateral Lucidis Toric IOL implantation. The primary endpoint was binocular uncorrected distance visual acuity (UDVA) at 3 months. Secondary endpoints included monocular/binocular UDVA, distance-corrected VA (DCVA) at 4 m; intermediate (80 cm, 66 cm) and near (40 cm) uncorrected and distance-corrected acuities; defocus curve; residual spherical equivalent (SE) and cylinder; IOL rotation; optical quality (MTF cutoff, Strehl ratio, HOA RMS); contrast sensitivity; halometry; and NEI-RQL-42. Paired *t*-tests or Mann–Whitney tests compared pre-/postoperative values.

**Results:**

At 3 months, the mean postoperative spherical equivalent was 0.17 ± 0.52 D (median 0.00 D; range −0.75 to +0.75 D) and the mean refractive cylinder was 0.04 ± 0.32 D (median 0.00 D; range −0.50 to +0.50 D). Median IOL rotation was 2.1 ± 2.3 degrees, with no eyes requiring repositioning. Mean binocular UDVA, UI80VA, UI66VA and UNVA were −0.01 ± 0.1, 0.08 ± 0.24, 0.04 ± 0.1, and 0.01 ± 0.14 logMAR, respectively. There were no statistically significant differences between uncorrected and distance-corrected visual acuities at any distance. The binocular defocus curve showed visual acuity better than 0.1 logMAR from +0.50 D to −3.00 D. NEI-RQL-42 scores indicated high patient satisfaction, particularly in clarity of vision, far, near vision, activity limitations, and glare domains.

**Conclusion:**

Bilateral Lucidis Toric EDOF IOL implantation delivers stable rotational performance, broad-range uncorrected vision, and high spectacle independence in astigmatic cataract patients. Future randomised, head-to-head trials with longer follow-up are warranted.

## 1 Introduction

Phacoemulsification with intraocular lens (IOL) implantation is the most commonly performed ophthalmic procedure worldwide; it restores vision by replacing the opacified crystalline lens with an artificial optic. Conventional monofocal IOLs deliver high-quality vision at a single focal point, but patients typically require spectacles for either near or distance tasks (1, 2). As life expectancies rise and visual demands broaden, there is growing interest in premium IOLs that extend uncorrected, spectacle-independent vision across multiple distances (2).

Premium IOLs fall into several optical categories: accommodative, multifocal, extended range of vision (ERV), and extended depth-of-focus (EDOF). Accommodative lenses attempt to mimic the eye’s natural focus shift but often provide only limited near acuity while diffractive multifocal IOLs split incoming light into discrete foci (e.g., distance and near), trading off contrast and risking photic phenomena such as halos and glare (3, 4). In contrast, ERV and EDOF designs aim to create a continuous through-focus profile without distinct gaps. Diffractive EDOF optics employ echelette gratings to subtly reshape the wavefront, whereas refractive EDOF lenses utilize a refractive design to elongate the focal point, creating a broader range of clear vision from far to intermediate distances (5–7).

Uncorrected corneal astigmatism remains a critical limiter of postoperative visual function, reducing contrast and disrupting extended-focus performance even at low-to-moderate levels (≥ 0.75 D) (8, 9). Toric IOLs that combine cylindrical correction with EDOF optics are therefore essential for astigmatic cataract patients. To date, clinical and bench evaluations have focused on the non-toric Lucidis IOL which integrates the EDOF technology Instant Focus built into its aspheric optical center surrounded by its refractive outer surface, demonstrating broad through-focus performance but leaving unanswered how the toric element affects optical quality and patient outcomes (10–12).

This prospective study fills that gap by assessing visual acuity (distance, intermediate, near), refractive accuracy, rotational stability, optical quality metrics, and patient-reported satisfaction at 3 months after bilateral implantation of the Lucidis Toric EDOF IOL in patients with regular corneal astigmatism.

## 2 Materials and methods

All participants provided written informed consent under protocol 2,979 CESC, approved by the University Hospital Ethics Committee, in accordance with the Declaration of Helsinki.

The authors declare that the research was conducted in the absence of any commercial or financial relationships that could be construed as a potential conflict of interest.

### 2.1 Study design and population

This study enrolled patients scheduled for bilateral cataract surgery who had regular corneal astigmatism and sought spectacle independence. Inclusion criteria were: bilateral cataract; age ≥ 18 years; regular corneal astigmatism 1.00–3.00 D in both eyes by tomography; willingness to attend all postoperative visits; absence of non-cataract media opacities; IOL power between +14.00 and +26.00 D; and written informed consent. Exclusion criteria included: irregular or asymmetric corneal astigmatism; any corneal pathology; uncontrollable dry eye; glaucoma or optic neuropathies; retinal/macular disease reducing acuity < 0.3 logMAR or contrast; planned secondary ocular surgery (except Nd: YAG capsulotomy); prior corneal refractive surgery; capsular or zonular abnormalities (e.g., pseudoexfoliation, uveitis, Marfan etc.); insufficient capsular support; pupillary abnormalities; amblyopia; or any other condition likely to impair visual gain.

Eligible subjects underwent standard cataract extraction using phacoemulsification and continuous curvilinear capsulorhexis, leaving the posterior capsule intact. All patients received bilateral implantation of the Lucidis Toric intraocular lens 12.4 mm (Lucidis, SAV-IOL, Route des Falaises 74, 2000 Neuchâtel, Switzerland), with the second eye operated within 7 days of the first. The IOL was targeted at emmetropia using the KANE formula for Toric IOLs, selecting the IOL power (D) allowing the first negative refractive value. The cylinder power with less residual cylinder was chosen (13). The IOL axis, calculated through the Kane formula for toric IOLs, was marked preoperatively while the patient was sitting at the slit-lamp looking at a distant target at head height with the fellow eye. Using the rotator switch, the slit light of the slit-lamp was just turned on to the IOL axis previously calculated. Then, two tips of that meridian were marked with a fine-tipped marking pen, where the slit light crossed at the limbus 180° away (14).

### 2.2 Clinical protocol

All patients underwent a comprehensive ophthalmic examination preoperatively and at 3 months post-implantation. Preoperative tests included uncorrected distance visual acuity (UDVA) and distance-corrected visual acuity (DCVA) at 4 m; uncorrected near visual acuity (UNVA) and distance-corrected near visual acuity (DCNVA) at 40 cm (CSO Vision Charts V14.0; CSO, Florence, Italy); corneal tomography (MS-39; CSO, Florence, Italy); optical biometry (Lenstar 900; Haag-Streit Diagnostics, Koeniz, Switzerland); Goldmann applanation tonometry; slit-lamp biomicroscopy; dilated fundus examination; and spectral-domain OCT (Spectralis OCT; Heidelberg Engineering, Heidelberg, Germany).

IOL stability was assessed at postoperative days 1 and 7, and again at 1 and 3 months; stability was defined as ≤5° rotation from the intended axis without residual cylinder >0.75 D, calculated via Toric IOL Assistant (Osiris T; CSO, Florence, Italy).

At 3 months, we measured UDVA, DCVA, UNVA, DCNVA, intermediate acuities at 80 cm and 66 cm (UI80VA, DC80VA, UI66VA, DC66VA), and mean refractive spherical equivalent (MRSE). Binocular defocus curves were obtained between +1.50 and −3.50 D using regular shifts of 0.50 D concerning the 4 m DCVA and recording the best visual acuity for each step. To avoid memory effects, presenting letter sequences was randomised, and patients’ eyes were occluded between each lens presentation (15).

Additional tests included contrast sensitivity under photopic (80 cd/m^2^), mesopic (6 cd/m^2^), and scotopic (3 cd/m^2^) conditions (CSV-1000 HGT; Vector Vision, Greenville, OH); aberrometry (RMS and Strehl ratio) via pyramidal wavefront (Osiris T Aberrometer; CSO, Florence, Italy); The objective ocular optical quality analysis included Root Means Square (RMS) and Point-Spread-Function Strehl ratio (PSF Strehl ratio), which is defined as the ratio between the peak image intensity of the patient’s eye and that of an ideal eye (i.e., maximal intensity), limited only by diffraction (16). Halo area was mapped using a centrally mounted LED (Golden Dragon Pluc LCW W5AM. PC, 5000 K; Osram, Munich, Germany) on an iPad4, moving 0.3 logMAR letters toward the light in 0.05° steps; patients at 2 m in darkness identified letters along six meridians, recording the cut-off angle in each direction (17). Patient quality of life was assessed via the NEI-RQL-42, comprising 13 subscales and 42 items across 16 response formats (18). Posterior capsular opacification ≥ Grade 3 was treated with YAG capsulotomy, and the 3-month visit was deferred by 10 days (19).

### 2.3 IOL description

The Lucidis Toric IOL is a single-piece, EDOF aspheric lens based on aberrometric technology. It features closed-loop haptics and a 360° square-edge design, with an optical diameter of 6.0 mm and a total diameter available in two sizes: 10.8 mm and 12.4 mm. The lens is constructed from hydrophilic acrylic material with a water content of 26% (12).

The Lucidis design uses a multizone refractive-aspheric profile combining both refraction and an aspheric element: a 1.0 mm central aspheric “axicon” zone generates a Bessel-beam focus for continuous intermediate-to-near vision, surrounded by a refractive ring supporting crisp distance optics (10). This beam starts to diverge only after a certain distance from the lens, thereby covering the entire range of vision from near-intermediate to far distance. The axicon-based central zone introduces no net spherical aberration; its surrounding refractive annulus up to the 6.0 mm optic edge enhances depth of focus while preserving high-quality distance imaging, all in a foldable design suited for micro-incision capsular-bag implantation. Available spherical powers span +5.00 to +30.00 D in 0.50 D steps, with toric cylinders of 1.00, 1.50, 2.25, 3.00, 3.75, or 4.50 D (12). According to the manufacturer’s documentations, the benefit of this particular design is to provide some degree of near and intermediate vision compared to classic monofocal optics calculated for emmetropia, while achieving the same optical qualities and visual acuity for far vision, and being aberrations-neutral to keep the rate of dysphotopsia to a minimum.

### 2.4 Statistical analysis

Statistical analyses were performed using IBM SPSS Statistics version 24 (IBM-SPSS). Normality of continuous variables was assessed with the Shapiro–Wilk test. Parametric data are presented as mean ± standard deviation; non-parametric data as median (interquartile range). Paired comparisons used the paired t-test for normally distributed data and the Wilcoxon signed-rank test for non-normally distributed data. A two-tailed *p* < 0.05 was considered statistically significant.

Based on an expected 0.5-logMAR loss in near vision per 2.00 D astigmatism and assuming a DCNVA of 0.01 ± 0.10 logMAR, 18 patients were needed for 90% power at *α* = 0.01. To allow for dropouts, 25 patients were recruited. Subjects with intraoperative complications or incomplete follow-up were excluded from analysis.

## 3 Results

Fifty eyes from 25 patients (nine men, 16 women) underwent bilateral cataract surgery with implantation of the toric Lucidis IOL. All patients completed the 3-month follow-up, and none had intraoperative complications. The mean age of the enrolled patients was 67.5 ± 3.8 years. Preoperatively, mean axial length was 23.75 ± 1.6 mm and mean corneal keratometry was 44.52 ± 1.4 D (median 44.43 D; range 41.30–47.02 D). Mean corneal astigmatism targeted for correction was 1.27 ± 0.22 D (median 1.27 D; range 1.07–2.96 D). The implanted IOL had a mean spherical dioptric power of 18.25 ± 4.34 D (median 18.50 D; range 14.0–26.0) and a mean toricity of 1.56 ± 0.05 D (median 1.50 D; range 1.00–4.50 D).

All patients completed 3 months of follow-up without intraoperative complications. At that visit, mean rotation was 2.1 ± 2.3° (range 0.1–4.6°), and PCO grade 3–4 was noted in 5 eyes (10%), which underwent YAG capsulotomy. The MRSE at 3 months was 0.17 ± 0.52 D (median 0.00 D; range −0.75 to +0.75 D), with a mean refractive cylinder of 0.04 ± 0.32 D (median 0.00 D; range −0.50 to +0.50 D).

### 3.1 Visual outcomes

**Figure 1:**
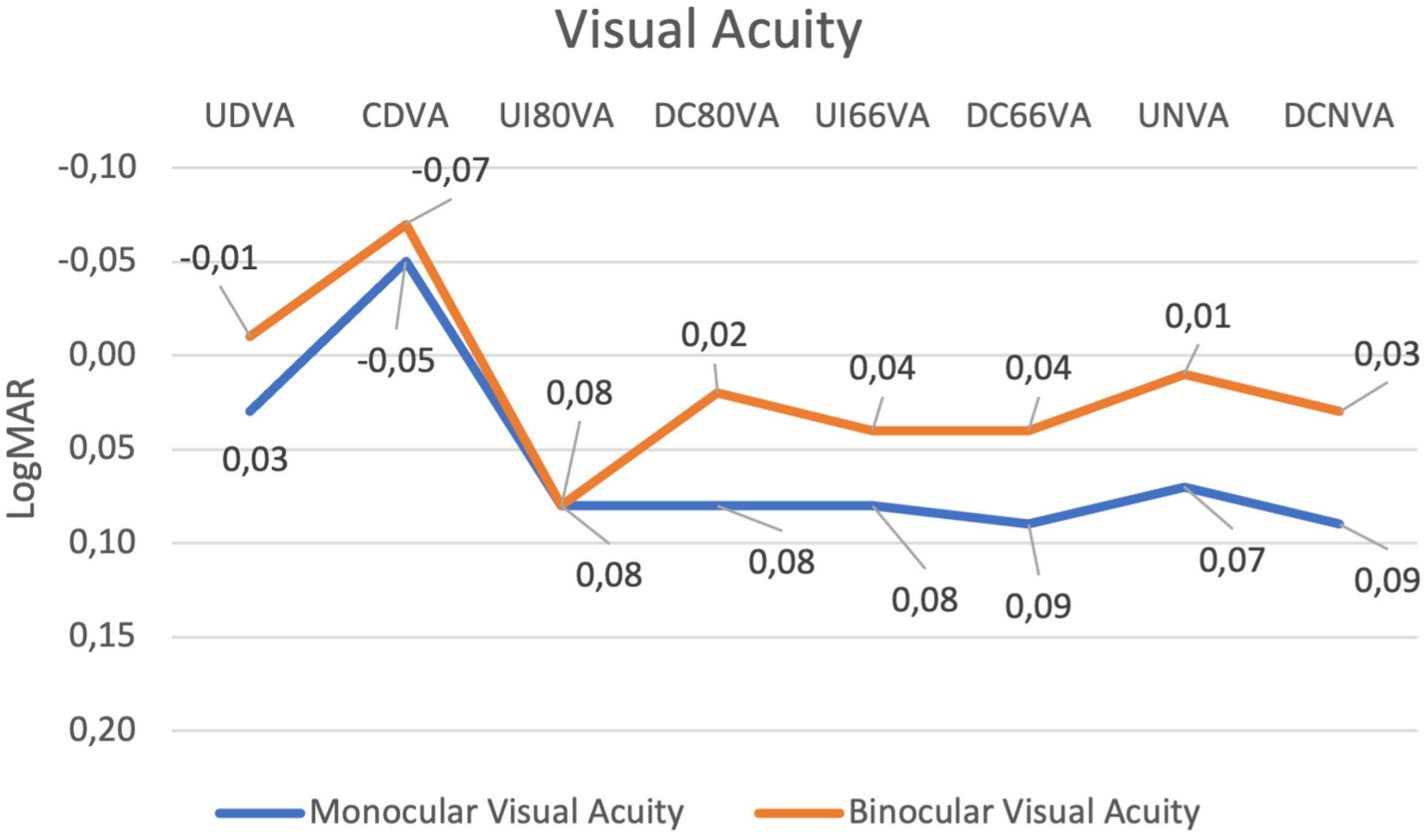
Line graph showing changes in monocular and binocular visual acuity across various conditions measured in LogMAR. The blue line represents monocular visual acuity, and the orange line represents binocular visual acuity. Both data sets fluctuate, with minimal peaks and dips at different points, highlighting variations in visual performance with overall good performance with all distances.

Table 1 summarises the visual acuity outcomes. The differences between corrected and uncorrected visual outcomes were not statistically significant for all of the studied distances (Figure 1).

**Table 1:** Monocular and binocular visual acuity outcomes 3 months post-operatively.

Visual acuity	Monocular vision			*p-*value	Binocular vision			*p-*value
	Mean ± SD	Median (range)	Confidence interval 95%		Mean ± SD	Median (range)	Confidence interval 95%	
UDVA (4 m)	0.03 ± 0.12	0.00 (−0.20 to 0.20)	0.00–0.07	0.2	−0.01 ± 0.10	0.00 (−0.20 to 0.20)	−0.06 to 0.04	0.16
CDVA (4 m)	−0.05 ± 0.17	0.00 (−0.20 to 0.20)	−0.10 to 0.00		−0.07 ± 0.08	−0.10 (−0.20 to 0.10)	−0.11 to −0.03	
UIVA (80 cm)	0.08 ± 0.13	0.00 (−0.10 to 0.40)	0.04–0.12	0.39	0.08 ± 0.24	0.00 (−0.10 to 0.40)	−0.03 to 0.20	0.1
DCIVA (80 cm)	0.08 ± 0.13	0.04 (−0.10 to 0.40)	0.04–0.12		0.02 ± 0.10	0.00 (−0.10 to 0.30)	−0.03 to 0.06	
UIVA (67 cm)	0.08 ± 0.14	0.00 (−0.10 to 0.40)	0.04–0.13	0.49	0.04 ± 0.10	0.00 (−0.10 to 0.40)	−0.01 to 0.09	0.13
DCIVA (67 cm)	0.09 ± 0.12	0.10 (−0.10 to 0.40)	0.05–0.13		0.04 ± 0.11	0.00 (−0.10 to 0.40)	−0.01 to 0.10	
UNVA (40 cm)	0.07 ± 0.13	0.00 (−0.10 to 0.40)	0.03–0.11	0.35	0.01 ± 0.14	0.00 (−0.20 to 0.40)	−0.06 to 0.08	0.14
DCNVA (40 cm)	0.09 ± 0.12	0.00 (−0.10 to 0.40)	0.05–0.12		0.03 ± 0.13	0.00 (−0.20 to 0.40)	−0.03 to 0.09	

Uncorrected (UDVA/UIVA/UIVA/UNVA) and distance-corrected (CDVA/DCIVA/DCIVA/DCNVA) visual acuities were measured at distance (4 m), intermediate (80 cm and 67 cm), and near (40 cm). Data are presented as mean ± SD, median (range) and confidence interval. *p*-values (paired *t*-test or Mann–Whitney test) compare uncorrected versus distance-corrected acuities; no differences reached statistical significance (all *p* > 0.05).

### 3.2 Defocus curve outcomes

**Figure 2:**
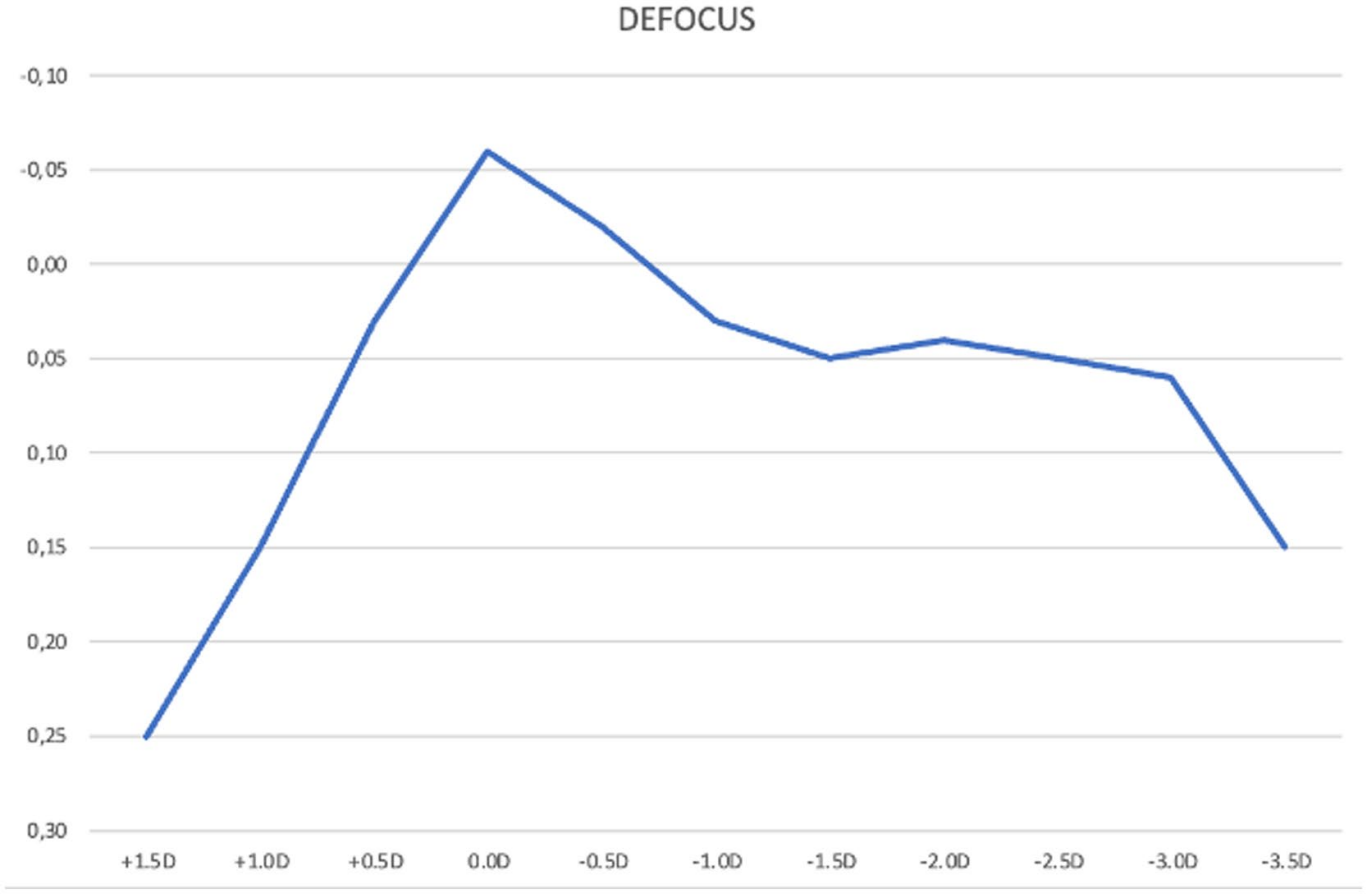
Mean binocular best corrected defocus curve at 3 months post-implantation of the Lucidis Toric EDOF IOL. The x-axis in the graph represents defocus power lenses added to the best correction at 4 m; the y-axis indicates visual acuity in LogMAR.

The mean binocular defocus curve demonstrated that visual acuity remained functionally good across a wide range of vergences. Visual acuity remained better than 0.2 logMAR across vergences from +1.00 D to −3.50 D at all tested defocus steps, indicating a broad range of usable vision. Notably, from +0.50 D to −3.00 D, visual acuity was equal to or better than 0.1 logMAR, suggesting effective depth of focus for both intermediate and near vision. Peak performance occurred at plano (0.0 D) with a mean logMAR of −0.06 (better than 20/20 Snellen). Beyond −3.00 D, acuity declined to 0.15 logMAR at −3.50 D, reflecting a gradual taper rather than an abrupt drop-off and confirming the IOL’s extended depth-of-focus profile (Figure 2).

### 3.3 Ocular optical quality outcomes

The mean postoperative modulation transfer function (MTF) cut-off was 18.03 ± 3.99 cycles/degree (cpd; median 17.8; range 16.3–38.6 cpd), and the mean Strehl ratio was 0.19 ± 0.08 (median 0.18; range 0.06–0.34). Mean higher-order aberration (HOA) root-mean-square (RMS) was 0.22 ± 0.16 μm (median 0.23; range 0.09–0.92 μm).

### 3.4 Halometry

**Figure 3:**
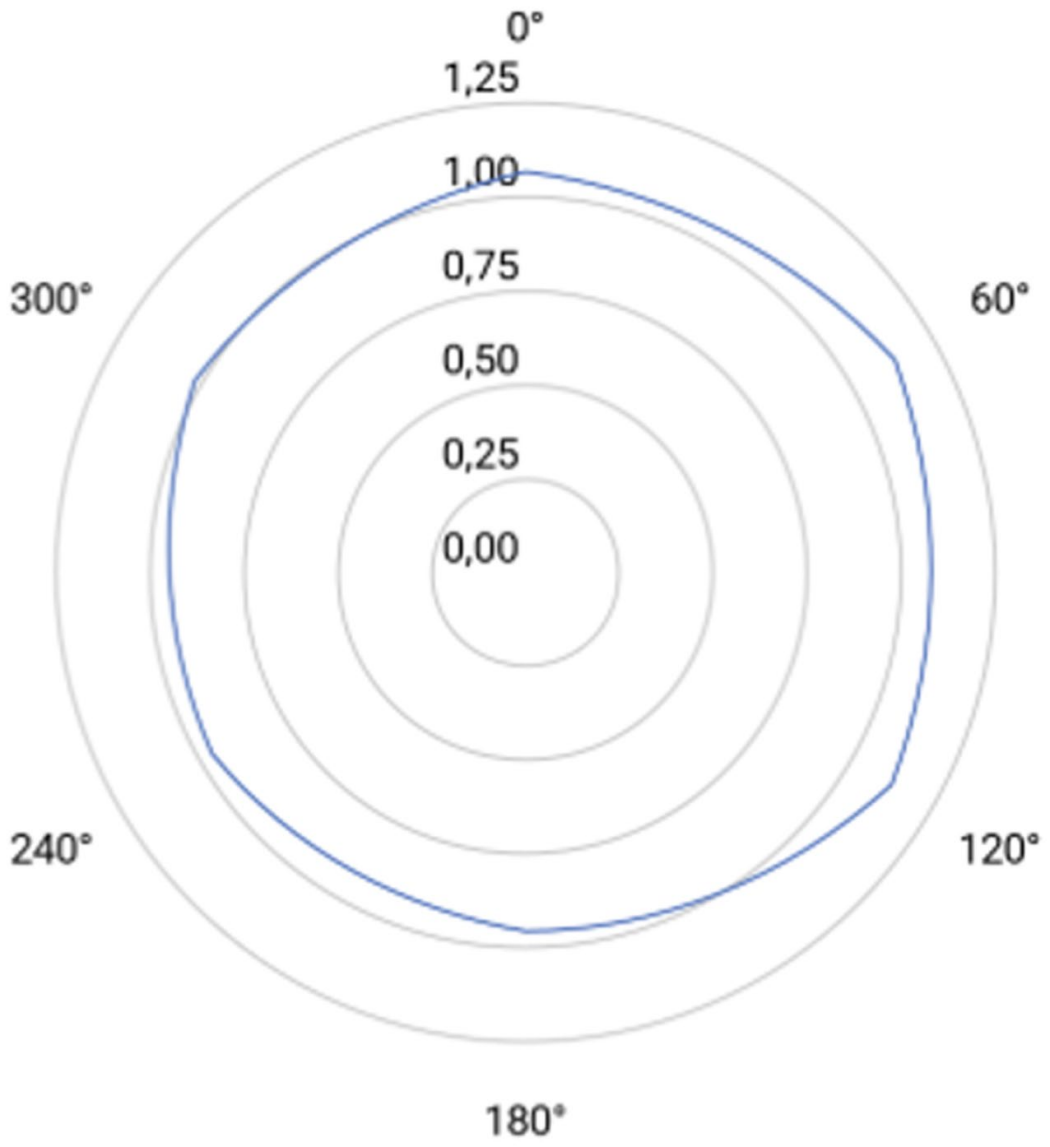
Polar plot of halometric measurements taken at six meridians (0°, 60°, 120°, 180°, 240° and 300°). Concentric circles indicate radial increments of 0.25 mm (from 0.00 to 1.25 mm). Light-blue line rappreen the post-implantation mean halometric radius at each meridian.

Halometry revealed slight variations in halo radius across meridians. The largest mean radii occurred at 60° and 120° (1.13 mm), and the smallest at 180° (0.96 mm), with intermediate values at 0° (1.06 mm), 240° (0.97 mm), and 300° (1.01 mm). Mean binocular halo radii are plotted in Figure 3. No statistically significant differences were observed among the values, except between the results at 60° and 180° (*p* = 0.023).

### 3.5 Contrast sensitivity outcomes

**Figure 4:**
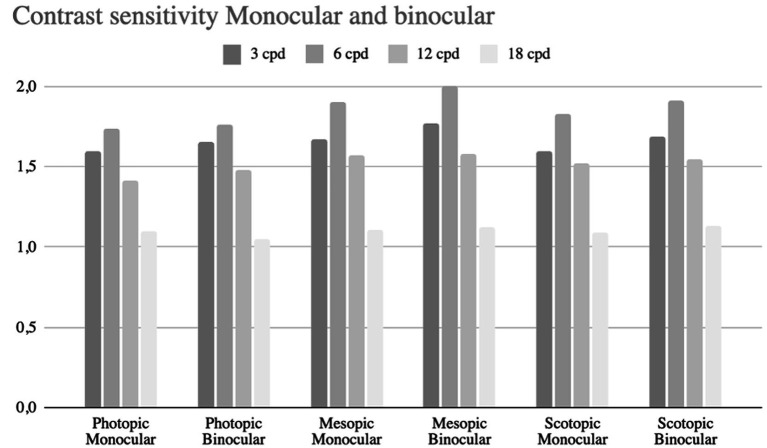
Bar chart comparing contrast sensitivity under photopic, mesopic, and scotopic conditions for monocular and binocular vision. Sensitivity is measured at four spatial frequencies: 3, 6, 12, and 18 cycles per degree (cpd).

Binocular and monocular contrast sensitivity were measured under photopic (80 cd/m^2^), mesopic (6 cd/m^2^), and scotopic (3 cd/m^2^) conditions. Photopic contrast sensitivity did not differ significantly from mesopic or scotopic values (*p* > 0.05), nor between binocular and monocular measurements (Figure 4).

### 3.6 Quality of life outcomes

Postoperative quality of life outcomes evaluated with the NEI RQL-42 questionnaire were summarised in 13 domains (Table 2).

**Table 2:** Postoperative NEI-RQL-42 questionnaire scores at 3 months.

Parameter	Mean ± SD	Median (range)
Clarity of vision	74.42 ± 30.05	100 (0–100)
Expectations	30.77 ± 42.61	0 (0–100)
Near vision	77.66 ± 27.79	75 (0–100)
Far vision	86.51 ± 23.89	100 (0–100)
Diurnal fluctuation	61.75 ± 33.76	66 (0–100)
Activity limitations	92.86 ± 20.06	100 (0–100)
Glare	85.00 ± 29.58	100 (0–100)
Symptoms	71.95 ± 27.68	75 (0–100)
Dependence on correction	74.67 ± 34.06	100 (0–100)
Worry	46.67 ± 35.80	50 (0–100)
Suboptimal correction	98.21 ± 6.56	100 (75–100)
Appearance	78.70 ± 34.96	100 (0–100)
Satisfaction with correction	78.67 ± 25.60	80 (0–100)

Mean ± SD and median (range) are shown for each of the 13 domains of the National Eye Institute Refractive Error Quality-of-Life Instrument (NEI-RQL-42); scores range from 0 (worst) to 100 (best).

## 4 Discussion

Residual corneal astigmatism continues to be a key limiting factor in achieving optimal outcomes in premium cataract surgery, and its correction is essential (20). Untreated astigmatism has been shown to reduce distance visual acuity by approximately 0.10 logMAR for each diopter (8, 9).

In our cohort, the Lucidis Toric IOL proved to be safe and effective in correcting astigmatism, also showing excellent stability over 3 months; indeed, no eye required surgical repositioning due to IOL rotation, indicating that the observed rotation remained within clinically acceptable limits. It must be emphasised that these reports refer only to the 12.4-diameter IOL. We cannot comment on the 10.8 mm version, but it is conceivable that the larger version may provide greater stability, especially in myopic eyes. The closed-loop haptic design and hydrophilic acrylic material likely contribute to this stability within the capsular bag (21).

In this prospective series, bilateral implantation of the Lucidis Toric EDOF IOL yielded excellent visual outcomes, with patients showing mean CDVA between −0.07 and 0.04 logMAR at all the tested distances. To the best of our knowledge, this is the first study to report the visual performance of the toric version of the Lucidis extended depth-of-focus intraocular lens. These results were, furthermore, confirmed at the defocus curve, where between +0.50 D and −3.00 D it was equal to or better than 0.1 logMAR.

Rabinovich et al. reported binocular UDVA of 0.038 ± 0.05 logMAR, UIVA of 0.09 ± 0.10 logMAR, and UNVA of 0.16 ± 0.14 logMAR following implantation of the non-toric Lucidis IOL. The high performance of the toric Lucidis model is notable also in monocular. Our results show monocular UDVA of 0.03 ± 0.12 logMAR, UIVA80 of 0.08 ± 0.13 logMAR, UIVA67 0.08 ± 0.14 logMAR and UNVA of 0.07 ± 0.13 logMAR. Indeed, Aref et al. found monocular UDVA ≈ 0.20 logMAR, UIVA ≈ 0.07 logMAR, and UNVA ≈ 0.15 logMAR (10, 11).

Although no direct comparative study has been conducted, the visual performance of the toric Lucidis IOL appears to be superior when considered in the context of published outcomes for its non-toric counterpart (10, 11). In fact, in our cohort, the mean preoperative corneal astigmatism was 1.27 ± 0.22 D (median 1.27 D; range 1.07–2.96 D), which could have resulted in a potential visual acuity loss of 0.1 to 0.2 logMAR. The implantation of the toric Lucidis IOL, in our cohort, effectively neutralised the corneal cylinder, reducing residual refractive astigmatism to 0.04 ± 0.32 D (median: 0.00 D; range: −0.50 to +0.50 D) explaining possibly the higher visual acuity when compared with the non-toric IOL as it was reported by the authors (10).

However, the influences on the visual performance related to the presence of the toricity cannot be ruled out in this study. An optical bench assessment of the toric model would be valuable for clarifying the influence of the toric element on the lens’s performance characteristics, giving the fact that until now, only the performance of the non-toric version has been analysed (22).

A key finding was that uncorrected and distance-corrected visual acuities did not differ significantly at any tested distance (all *p* > 0.05; Table 1), underscoring excellent spectacle independence. This “forgiving” performance aligns with prior reports that EDOF optics maintain ≥20/25 visual acuity despite up to ±1.00 D of spherical defocus, whereas multifocal designs degrade beyond ±0.50 D of defocus (23, 24). Consequently, in our cohort, only 2 eyes with a postoperative spherical equivalent exceeding ±0.50 D showed clinically meaningful improvements in visual acuity following spectacle correction.

Indirectly comparing our results with the visual outcomes of a toric ERV, both toric IOLs demonstrate similar performance for distance and intermediate vision; however, the pseudo–non diffractive design provides superior results for near vision (mean binocular UNVA 0.09 logMAR vs. 0.00 logMAR) (25). Moreover, although the median IOL rotation was similar, two patients in that cohort of 30 required re-centering, whereas none of our patients underwent further surgery, suggesting a potentially higher stability in our group.

Patient-reported outcomes further confirmed these findings. The NEI RQL-42 questionnaire revealed high satisfaction across multiple domains: Activity Limitations (mean ± SD, 92.86 ± 20.06), Far Vision (86.51 ± 23.89), Dependence on Correction (74.67 ± 34.06), and Near Vision (77.66 ± 27.79) (Table 2). These elevated scores underscore the high level of spectacle independence and align with our measured uncorrected acuities at near, intermediate, and distance. Moreover, these results closely mirror those from our prior evaluation of the non-toric Lucidis IOL (26).

Unfortunately, neither Rabinovich et al. nor Aref et al. reported patient-reported quality-of-life outcomes and different cohorts in other studies prevent direct comparisons with other EDOF IOL data. (10, 11, 20, 27, 28). However, our visual-quality metrics and NEI-RQL-42 scores closely mirror those in published series, suggesting similarly high patient-perceived vision quality (20, 27, 28).

Moreover, the Lucidis Toric IOL demonstrated objective optical performance comparable to its non-toric version and other premium lenses, with an MTF cut-off of 18.03 ± 3.99 cpd/deg., a Strehl ratio of 0.19 ± 0.08, and an HOA RMS of 0.22 ± 0.16, indicating minimal photic disturbances in toric configuration (27). For instance, the non-toric platform exhibited a mean PSF Strehl ratio of 0.20 ± 0.09, with mean ocular and corneal RMS values at 4 mm of 0.31 ± 0.28 and 0.19 ± 0.07, respectively (26). Both IOLs demonstrated a higher Strehl ratio compared to the continuous transition of focus IOL (Precizon Presbyopic IOL), which showed a mean Strehl ratio of 0.11 ± 0.07. The MTF cut-off point values were nearly comparable between the studied IOL and the Precizon Presbyopic, measuring 20.03 ± 4.86 cpd/deg (25).

The present study has several limitations. First, its non-randomized design with no control prevents direct comparisons with other IOL types, such as non-toric monofocal or EDOF models, which limits the generalizability of our conclusions. Second, the relatively small sample size (25 patients) may reduce the statistical power and increase the risk of type II error, especially in subgroup analyses (i.e., by cylinder magnitude or age). Third, the follow-up period was limited to 3 months, which does not allow assessment of the long-term stability of visual outcomes, rotational stability of the toric component, posterior capsular opacification rate, or late postoperative complications. Fourth, we did not stratify results by pupil size, angle kappa, or other biometric factors known to affect EDOF IOL performance; this enhances generalizability but reduces reproducibility. Finally, although we included objective aberrometry and contrast-sensitivity testing, we did not evaluate postoperative tolerance to induced astigmatism, which is critical for Toric EDOF optics. Future randomized controlled trials with larger cohorts and extended follow-up will be necessary to validate these preliminary findings and to compare the performance of Lucidis Toric EDOF IOLs against other presbyopia-correcting technologies.

## 5 Conclusion

In conclusion, this first prospective evaluation of the Lucidis Toric EDOF IOL demonstrates excellent rotational stability (mean rotation 2.1° ± 2.3°), broad-range uncorrected visual acuity (better than 0.1 logMAR from +0.50 D to −3.00 D), and high spectacle independence. Patient-reported outcomes confirm strong satisfaction across functional domains. These findings support the Lucidis Toric as a safe and effective option for correcting presbyopia and astigmatism in cataract surgery, warranting further randomised, comparative studies.

## Data Availability

The raw data supporting the conclusions of this article will be made available by the authors, without undue reservation.

## References

[1] Schallhorn SC Hettinger KA Teenan D Venter JA Hannan SJ Schallhorn JM. Predictors of patient satisfaction after refractive lens exchange with an extended depth of focus IOL. J Refract Surg. (2020) 36:175–84. doi: 10.3928/1081597X-20200211-01, PMID: 32159822

[2] Pedrotti E Neri E Bonacci E Barosco G Galzignato A Montresor A. Extended depth of focus versus monofocal IOLs in patients with high myopia: objective and subjective visual outcomes. J Refract Surg. (2022) 38:158–66. doi: 10.3928/1081597X-20211220-01 35275002

[3] Fernández-Vega-Cueto L Madrid-Costa D Alfonso-Bartolozzi B Vega F Millán MS Alfonso JF. Optical and clinical outcomes of an extended range of vision intraocular lens. J Refract Surg. (2022) 38:168–76. doi: 10.3928/1081597X-20220104-01, PMID: 35275001

[4] Pedrotti E Chierego C Talli PM Selvi F Galzignato A Neri E. Extended depth of focus versus monofocal IOLs: objective and subjective visual outcomes. J Refract Surg. (2020) 36:214–22. doi: 10.3928/1081597X-20200212-01 32267951

[5] Savini G Balducci N Carbonara C Rossi S Altieri M Frugis N. Functional assessment of a new extended depth-of-focus intraocular lens. Eye. (2019) 33:404–10. doi: 10.1038/s41433-018-0221-1, PMID: 30266985 PMC6460699

[6] Pedrotti E Carones F Aiello F Mastropasqua R Bruni E Bonacci E. Comparative analysis of visual outcomes with 4 intraocular lenses: monofocal, multifocal, and extended range of vision. J Cataract Refract Surg. (2018) 44:156–67. doi: 10.1016/j.jcrs.2017.11.011 29587972

[7] Savini G Schiano-Lomoriello D Balducci N Barboni P. Visual performance of a new extended depth-of-focus intraocular lens compared to a distance-dominant diffractive multifocal intraocular lens. J Refract Surg. (2018) 34:228–35. doi: 10.3928/1081597X-20180125-01 29634837

[8] Hayashi K Manabe S Motoaki Y. Effect of astigmatism on visual acuity in eyes with a diffractive multifocal intraocular lens. J Cataract Refract Surg. (2010) 36:1323–9. doi: 10.1016/j.jcrs.2010.02.016, PMID: 20656155

[9] RN MN Pazo E Millar Z Richoz O Nesbit A Moore TC. Threshold limit of postoperative astigmatism for patient satisfaction after refractive lens exchange and multifocal intraocular lens implantation. J Cataract Refract Surg. (2016) 42:1126–34. doi: 10.1016/j.jcrs.2016.05.007 27531287

[10] Rabinovich M Ceresara G Aramburu Del Boz A Al Khatib D Crespe M Bovet J. Visual outcomes after implantation of Lucidis EDOF IOL. J Ophthalmol. (2022) 2022:5100861. doi: 10.1155/2022/5100861, PMID: 35669466 PMC9167139

[11] Aref A Gillmann K Mermoud A. Clinical and aberrometric evaluation of a new refractive intraocular lens with central extended depth-of-focus (Lucidis©). J Clin Ophthalmol Vis. (2019) 1:1.

[12] Swiss Advanced Vision SAV-IOLSA. Instant Focus IOL: InFo [Brochure]. Neuchâtel, Switzerland: SAV (2017).

[13] Li XY Liao X Lin J Lan CJ Tan QQ. Effect of optional biometric parameters in the Kane formula on intraocular lens power calculation. PLoS One. (2023) 18:e0289033. doi: 10.1371/journal.pone.0289033, PMID: 37616217 PMC10449110

[14] Bayramlar H Dag Y Karadag R Cakici O. An easy and practical method for toric intraocular lens implantation: marking corneal astigmatic axis at slit-lamp. Int Ophthalmol. (2017) 37:179–84. doi: 10.1007/s10792-016-0250-3, PMID: 27169419

[15] Gupta N Wolffsohn JS Naroo SA. Optimizing measurement of subjective amplitude of accommodation with defocus curves. J Cataract Refract Surg. (2008) 34:1329–38. doi: 10.1016/j.jcrs.2008.04.031, PMID: 18655984

[16] Alió JL D’Oria F Toto F Balgos J Palazón A Versaci F. Retinal image quality with multifocal, EDoF, and accommodative intraocular lenses as studied by pyramidal aberrometry. Eye Vis (Lond). (2021) 8:37. doi: 10.1186/s40662-021-00258-y, PMID: 34615549 PMC8496005

[17] Buckhurst PJ Naroo SA Davies LN Shah S Buckhurst H Kingsnorth A. Tablet app halometer for the assessment of dysphotopsia. J Cataract Refract Surg. (2015) 41:2424–9. doi: 10.1016/j.jcrs.2015.05.041, PMID: 26703492

[18] Nichols JJ Mitchell GL Saracino M Zadnik K. Reliability and validity of refractive error-specific quality-of-life instruments. Arch Ophthalmol. (2003) 121:1289–96. doi: 10.1001/archopht.121.9.1289, PMID: 12963612

[19] Congdon N Fan H Choi K Huang W Zhang L Zhang S. Impact of posterior subcapsular opacification on vision and visual function among subjects undergoing cataract surgery in rural China: study of cataract outcomes and up-take of services (SCOUTS) in the caring is hip project, report 5. Br J Ophthalmol. (2008) 92:598–603. doi: 10.1136/bjo.2007.126714, PMID: 18441169

[20] Bonacci E Pagnacco C Anastasi M De Gregorio A Marchini G Pedrotti E. Toric aberrometric extended depth of focus intraocular lens: visual outcomes, rotational stability, patients’ satisfaction, and spectacle independence. J Pers Med. (2025) 15:88. doi: 10.3390/jpm15030088, PMID: 40137404 PMC11943596

[21] Kaur M Shaikh F Falera R Titiyal JS. Optimizing outcomes with toric intraocular lenses. Indian J Ophthalmol. (2017) 65:1301–13. doi: 10.4103/ijo.IJO_810_17 29208810 PMC5742958

[22] Bang SP Jung HW Li KY Yoon G. Comparison of modal and zonal wavefront measurements of refractive extended depth of focus intraocular lenses. Biomed Opt Express. (2024) 15:1618–29. doi: 10.1364/BOE.513529 38495697 PMC10942709

[23] Braga-Mele R Chang D Dewey S Foster G Henderson BA Hill W. Multifocal intraocular lenses: relative indications and contraindications for implantation. J Cataract Refract Surg. (2014) 40:313–22. doi: 10.1016/j.jcrs.2013.12.011, PMID: 24461503

[24] Carones F. Residual astigmatism threshold and patient satisfaction with bifocal, trifocal and extended range of vision intraocular lenses (IOLs). Open J Ophthalmol. (2017) 7:1–7. doi: 10.4236/ojoph.2017.71001

[25] Gundersen KG. Rotational stability and visual performance 3 months after bilateral implantation of a new toric extended range of vision intraocular lens. Clin Ophthalmol. (2018) 12:1269–78. doi: 10.2147/OPTH.S173120, PMID: 30050279 PMC6056149

[26] Pedrotti E Bonacci E Kilian R Pagnacco C Anastasi M Ventura M. Quality of vision and outcomes after bilateral implantation of pseudo-non diffracting beam IOL. Front Med. (2023) 10:1085280. doi: 10.3389/fmed.2023.1085280, PMID: 36950509 PMC10025297

[27] Tañá-Rivero P Rodríguez-Carrillo MD Tañá-Sanz P Ruiz-Santos M Tañá-Sanz S. Clinical outcomes of trifocal toric intraocular lenses. Eur J Ophthalmol. (2023) 33:1773–85. doi: 10.1177/11206721231155047, PMID: 36788496

[28] Akahoshi T. Three patient-reported outcomes questionnaires in Japanese patients undergoing cataract surgery with trifocal IOL implantation. Clin Ophthalmol. (2024) 18:2521–9. doi: 10.2147/OPTH.S478292, PMID: 39246556 PMC11380849

